# Low serum hyaluronic acid levels associated with spontaneous HBsAg clearance

**DOI:** 10.1007/s10096-015-2467-x

**Published:** 2015-08-21

**Authors:** S. Harkisoen, J. E. Arends, A. van den Hoek, K. J. van Erpecum, G. J. Boland, A. I. M. Hoepelman

**Affiliations:** Department of Internal Medicine and Infectious Diseases, University Medical Center Utrecht, Heidelberglaan 100, P.O. Box 85500, F02.126, 3508 GA Utrecht, The Netherlands; Department of Infectious diseases, Public Health Service of Amsterdam (GGD), Amsterdam, The Netherlands; Department of Internal Medicine, Division of Infectious Diseases, Tropical Medicine and AIDS, Academic Medical Center, Amsterdam, The Netherlands; Department of Gastroenterology and Hepatology, University Medical Center Utrecht, Utrecht, The Netherlands; Department of Medical Microbiology, University Medical Center Utrecht, Utrecht, The Netherlands

## Abstract

**Purpose:**

The pathophysiological underlying mechanism of spontaneous HBsAg clearance in hepatitis B virus (HBV) infected patients is largely unknown. However, serum hyaluronic acid (sHA) plays a role in liver fibrosis progression and reversely could serve as a potential biomarker for HBsAg clearance. This study investigates whether low sHA is associated with HBsAg loss in non-Asian HBV patients.

**Methods:**

Non-Asian women living in Amsterdam with known chronic HBV infection between 1990–2003 were invited for a single follow-up visit at the Municipal Health Service Amsterdam between September 2011 to May 2012. Serum hyaluronic acid and liver stiffness measurement together with clinical evaluation, biochemical and virologic blood tests were performed.

**Results:**

Of the 160 women, HBsAg loss occurred in 38 (23 %) patients between diagnosis and follow-up. sHA levels were lower in HBsAg negative patients compared to HBsAg positive patients (14.5 [9.4–27.2] ng/mL vs 25.0 [12.3–42.5] ng/mL, *p* <0.01). A similar distinction in sHA between low and high HBV DNA was noted. sHA had a significant discriminatory ability to differentiate between HBsAg positive and HBsAg negative patients, (AUC 0.65 [95 % CI 0.55–0.75], *p* < 0.01). In multivariable analysis only sHA level was associated with HBsAg loss (OR 0.4 [0.2–0.9]). Finally, F3-F4 fibrosis (cut-off >8.1 kPa) was diagnosed in 3 % in HBsAg negative patients compared to 10 % in HBsAg positive patients (*p* = 0.15).

**Conclusion:**

Serum HA levels are lower in patients who experience spontaneous HBsAg loss compared to HBsAg positive patients.

## Introduction

Liver inflammation is the hallmark of hepatotropic viruses like hepatitis B virus (HBV). During this chronic hepatic inflammation, synthesis and turnover of the extracellular matrix (ECM) is modulated by several cytokines with serum hyaluronic acid (sHA) being one of its major ECM components [1, 2]. In healthy persons, sHA levels vary between 23 and 129 ng/mL [3, 4]. sHA levels have already been shown to be higher in patients with chronic hepatitis C or active autoimmune hepatitis than those of healthy individuals without signs of hepatitis [5–7]. In addition, in chronic HBeAg negative HBV patients, high serum sHA positively correlates with fibrosis progression and level of inflammation [8]*.* Based on these observations, sHA level might be a reflection of the intensity of the immune response in chronic liver diseases.

Annually, approximately 2 % of all chronic hepatitis B virus (HBV) infected patients experience HBV surface antigen (HBsAg) loss without antiviral therapy (i.e. spontaneous HBsAg clearance) [9]. Although the exact pathophysiological mechanism of spontaneous HBsAg loss in patients with chronic HBV has not been fully understood, there is some evidence that a T-cell mediated immune response may be essential in this process [10]. Rehermann et al. [11] demonstrated that chronic HBV patients who cleared HBsAg, spontaneously or after interferon-alfa treatment, had a strong HBV multispecific cytotoxic T-lymphocyte response that was similar to those clearing HBsAg after an acute infection*.* Several histological studies have shown that after spontaneous HBsAg loss, liver inflammation declined over time [12, 13]*.* However, it is unknown whether sHA levels also decline after spontaneous HBsAg loss and if sHA could serve as a potential biomarker for HBsAg clearance. The aim of this study was to investigate whether a low sHA level is associated with HBsAg loss in patients with chronic HBV.

## Materials and methods

### Patient selection

The study population has already been described previously [14]. Briefly, non-Asian women in the greater Amsterdam area, who were registered as chronic HBV patients between 1990 and 2004, were invited for a single study visit between September 2011 and May 2012 at the Municipal Health Service of Amsterdam. History taking and physical examination were performed, along with a liver stiffness measurement (LSM) and blood tests to determine the hyaluronic acid level and biochemical and virological tests. The following biochemical tests were determined: bilirubin, alanine aminotransferase (ALT), aspartate aminotransferase (AST), Gamma-glutamyl transpeptidase (GGT), alkaline phosphatase (ALP) and albumin. For this study 14 patients were excluded from the 174 included patients (three with HCV co-infection, five with previous HBV treatment, one with acute HBV infection and five due to indeterminate sHA which was a result of not enough serum to determine the sHA), resulting in a study population of 160 participants.

Spontaneous HBsAg loss was defined as having a positive HBsAg antigen in the historic sample with subsequent HBsAg negativity during the follow-up visit without previously being subjected to antiviral therapy. The study protocol was approved by the Ethical Committee of the Academic Medical Center Amsterdam. Written approval was obtained from the Public Health Service Amsterdam. Informed consent was obtained from each participant (clinical trials number NCT01462981).

### Laboratory tests

Hyaluronic acid levels were measured in serum at the follow-up visit as part of the ELF-test by the ADVIA Centaur XP (Siemens, Erlangen, Germany). This assay has a lower detection limit of 1.6 ng/ml, a linear range from 1.6 to 1000 ng/ml, and higher concentrations are expressed as >1000 ng/ml. Linearity, sensitivity and precision were evaluated according to CLSI guidelines. HBV DNA was determined with the COBAS Ampliprep/COBAS Taqman assay, v.2.0 (Roche Molecular Diagnostics, California, USA) with a lower detection limit of 20 IU/ml. Qualitative anti-HCV, anti-HIV and qualitative HBsAg were also performed with the ADVIA Centaur XP assay. Lastly, liver-related biochemical parameters were determined according to local standard laboratory procedures. HBV DNA levels were divided in more or less than 2000 IU/mL that was derived from current international guidelines [15, 16].

### Liver stiffness measurement

An experienced researcher (S.H.) performed the LSM with a Fibroscan® (model F402 Echosens, France) according to standard operating procedures supplied by the manufacturer and as described by others [17]. The METAVIR classification was used to determine the fibrosis stage, categorizing no to mild fibrosis (F0/F2) with a score less than or equal to 8.1 kPa and severe fibrosis to cirrhosis (F3/F4) with a score above 8.1 kPa based on a cut-off value from previous studies in chronic HBV patients [18, 19].

### Statistical analysis

Continuous variables were expressed as a median with interquartile range (IQR) and categorical variables as frequencies with percentage. Differences between HBsAg positive and HBsAg negative groups were calculated with the Mann–Whitney U test (continuous variables) or chi-square test (categorical variables). Factors associated with HBsAg loss were analysed with univariable and multivariable logistic regression. Factors with a p-value < 0.10 in univariable analysis were included in the multivariable logistic regression. Correlation between ALT levels and sHA levels were calculated with the Spearman rho correlation. A receiver-operator characteristic (ROC) curve was constructed to assess the value of sHA in discriminating HBsAg positive patients from HBsAg negative patients and to determine an optimal cut-off value for the sHA. A ROC curve with an area under curve (AUC) less than 0.60 and a p-value >0.05 was considered unreliable for the ROC curve. Outcomes were reported as odds ratio (OR) with 95 % confidence intervals (CI), and a p-value <0.05 was considered significant. The statistical analysis was performed with SPSS v17 (version 17.0; SPSS Inc., Chicago, IL, USA).

## Results

Patient characteristics are given in Table 1. At the time of the follow-up study visit, HBsAg loss was documented in 38 (23 %) patients resulting in 122 HBsAg positive patients, all being HBeAg negative. All HBsAg negative patients had an undetectable HBV DNA level (i.e. <20 IU/ml). Of the 117 patients with a liver stiffness result, occurrence of F3-F4 fibrosis was lower in the HBsAg negative patients compared to HBsAg positive patients (one in 38 HBsAg negative patients (3 %) vs 12 in 122 HBsAg positive patients (10 %), *p* = 0.15).

**Table 1:** Characteristics of the study population by HBsAg status

Characteristic	HBsAg positive patients (N = 122)	HBsAg negative patients (N = 38)	p-value
Age, *years median (IQR)*	45 (41–49)	44 (39–48)	0.57
Follow-up, *years median (IQR)*	18 (13–20)	18 (14–20)	0.85
BMI, *kg/m²* *median (IQR)*	30 (27–33)	29 (25–34)	0.85
Alcohol, *n (%)* ^*a*^	39 (31)	5 (13)	0.02
Smoking, *n (%)* ^*b*^	28 (23)	3 (8)	0.03
Origin, *n (%)*			0.04
Turkey	33 (27)	19 (50)	
Ghana	31 (25)	3 (8)	
Surinam	25 (21)	5 (13)	
Morocco	24 (20)	7 (18)	
Other	9 (7)	4 (11)	
ALT, *U/L median (IQR)*	18 (15–26)	16 (12–21)	0.11
AST, *U/L median (IQR)*	17 (13–23)	15 (12–19)	0.16
Trombocytes, *10* ^*9*^ */L median (IQR)*	249 (201–287)	245 (218–284)	0.94
Protrombin time, *seconds median (IQR)*	13.1 (12.8–13.5)	13.2 (12.6–13.5)	0.82
Log HBV DNA*, IU/mL median (IQR)*	2.7 (1.9–3.5)	n/a	n/a
F3-F4 fibrosis, *n (%)* ^*c*^	12 (10)	1 (3)	0.15

*BMI* body mass index, *ALT* alanine aminotransferase, *AST* aspartate aminotransferase, *IQR* interquartile range, *U/L* units per liter, *IU/mL* international units per milliliter, *n/a* not applicable

^a^ Alcohol consumption defined as more than one glass per week

^b^ Smoking defined as more than one cigarette per week

^c^ Determined by liver stiffness measurement

### Hyaluronic acid level and HBV parameters

**Fig. 1:**
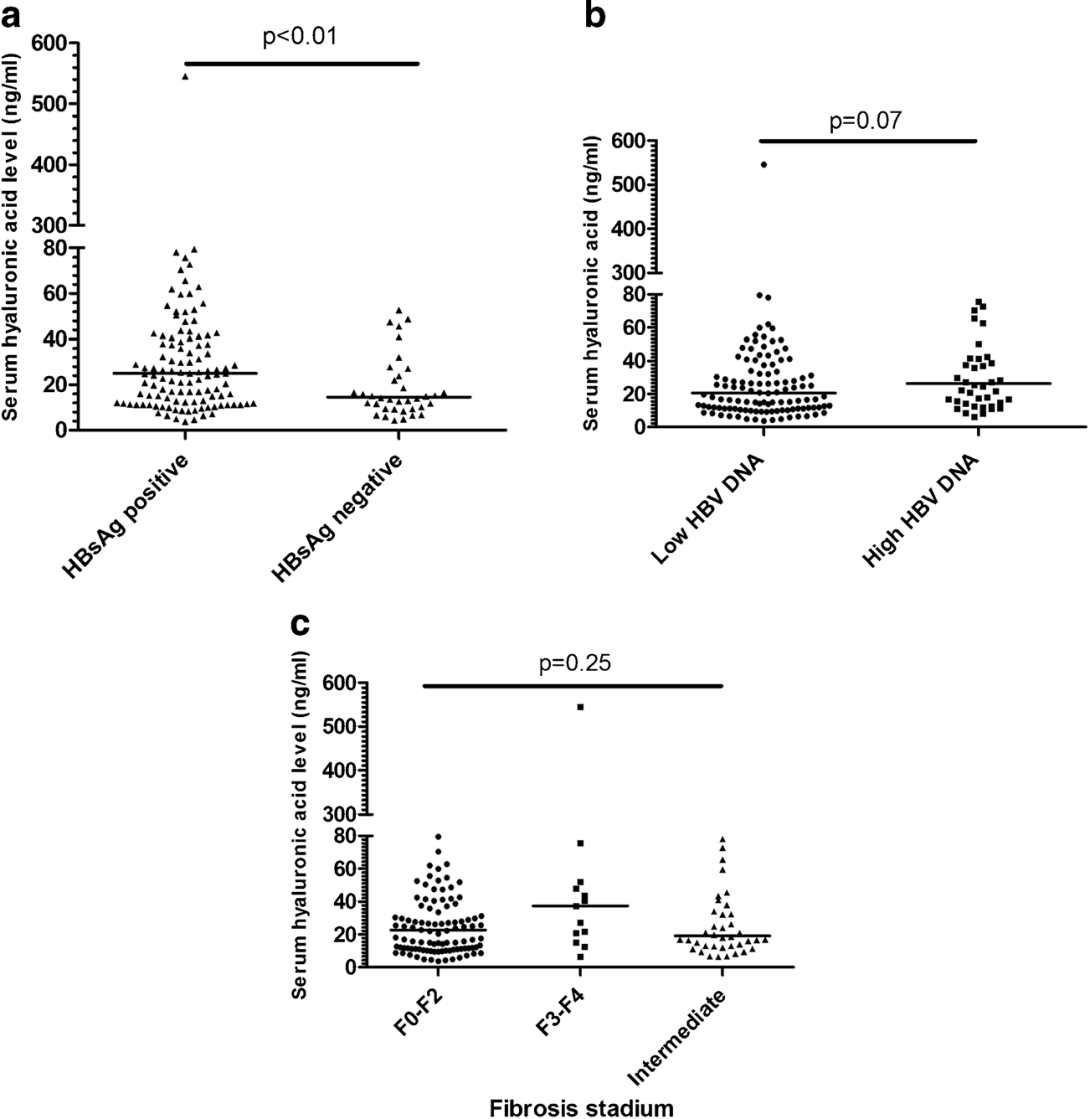
Distribution of serum hyaluronic acid according to HBsAg status (**a**), HBV DNA level (**b**) and fibrosis stage (**c**). *LSM* liver stiffness measurement

To explore whether sHA levels were different between HBsAg positive and HBsAg negative patients, sHA levels were compared in both groups. The median (IQR) sHA level of all included patients was 21.9 (11.7–41.2) ng/mL. HBsAg negative patients had a significantly lower sHA level compared to HBsAg positive patients (14.5 [9.4–27.2] ng/mL vs 25.0 [12.3–42.5] ng/mL, p <0.01) (Fig. 1). In addition, when patients were categorized into low HBV DNA level (HBV DNA less than or equal to 2000 IU/mL) and high HBV DNA level (HBV DNA > 2000 IU/mL), there was a trend towards lower sHA levels in the low HBV DNA patients compared to the patients with high HBV DNA (20.6 [11.2–40.4] ng/mL vs 26.3 [14.7–44.4] ng/mL, *p* = 0.07).

Overall, there was a positive correlation between sHA levels and ALT level in the whole study population (r = 0.20, p = 0.01) (data not shown). However, when patients were divided by HBsAg status, this correlation was lost (HBsAg positive group r = 0.16, *p* = 0.08 and HBsAg negative group, r = 0.11, *p* = 0.49). The sHA had a significant diagnostic discriminatory power to detect HBsAg negative patients (AUC 0.65 [95 % CI 0.55–0.75], *p* < 0.01). A cut-off value of 16.9 ng/mL was selected to have the optimum in both sensitivity and specificity. Subsequently, patients were divided in two groups, either low or high sHA. There were 68 (43 %) patients with a low sHA level compared to 92 (57 %) patients with a high sHA level. Of the 38 HBsAg negative patients, 25 (66 %) had a low sHA level (i.e. below 16.9 ng/mL) and 13 (34 %) a high sHA level.

### Factors associated with HBsAg loss

Next, we performed univariable and multivariable analyses to identify factors that were associated with HBsAg loss. The results are shown in Table 2. In univariable analysis, which included age, BMI, ethnic origin, smoking, alcohol consumption, ALT level, AST level, sHA level, HBV DNA and fibrosis stage as factors, alcohol consumption [OR 0.3 (0.1–0.9)] and a high sHA level [OR 0.3 (0.1–0.6)] were associated with increased chance to loose HBsAg. BMI, smoking, and ALT level had a close, but statistically not significant association with HBsAg loss [BMI OR 0.5 (0.2–1.1), smoking OR 0.3 (0.1–1.0) and ALT OR 0.5 (0.2–1.0)]. When all significant or borderline significant factors from univariable analysis were included in a multivariable analysis, only those patients with a high sHA level had a lower chance to lose HBsAg (OR 0.4 [0.2–0.8]).

**Table 2:** Analysis of factors associated with spontaneous HBsAg loss

Factor	Total (N)	HBsAg negative cases (%)	Univariable analysis OR (95 % CI)	p-value	Multivariable analysis OR (95 % CI)	p-value
Age						
≤ 45 years	85	22 (26)	1 (reference)	0.50	1 (reference)	
> 45 years	75	16 (21)	0.8 (0.4–1.6)			
BMI						
≤ 25 kg/m²	24	9 (38)	1 (reference)	0.09	1 (reference)	0.10
> 25 kg/m²	136	29 (21)	0.5 (0.2–1.1)		0.4 (0.2–1.2)	
Ethnic origin						
Turkey	52	19 (37)	1 (reference)	0.18	1 (reference)	
Ghana	34	3 (9)	1.3 (0.4–4.9)			
Surinam	30	5 (17)	0.2 (0.1–1.2)			
Morocco	31	7 (23)	0.5 (0.1–2.1)			
Other origin	108	19 (18)	0.7 (0.2–2.8)			
Smoking^a^						
No	129	35 (27)	1 (reference)	0.05	1 (reference)	0.06
Yes	31	3 (10)	0.3 (0.1–1.0)		0.3 (0.1–1.1)	
Alcohol consumption^b^						
No	116	33 (28)	1 (reference)	0.03	1 (reference)	0.17
Yes	44	5 (11)	0.3 (0.1–0.9)		0.5 (0.2–1.4)	
ALT						
≤ 0.5 x ULN	88	26 (30)	1 (reference)	0.06	1 (reference)	0.26
> 0.5 x ULN	72	12 (17)	0.5 (0.2–1.0)		0.6 (0.3–1.4)	
AST						
≤ 0.5 x ULN	150	38 (25)	1 (reference)		1 (reference)	
> 0.5 x ULN	10	0 (0)	n/a			
HA level						
Low	68	25 (37)	1 (reference)	<0.01	1 (reference)	0.01
High	92	13 (14)	0.3 (0.1–0.6)		0.4 (0.2–0.8)	
HBV DNA level						
Low/undetectable	118	38 (32)	1 (reference)		1 (reference)	
High	42	0 (0)	n/a			
Fibrosis stage						
F3-F4	13	1 (8)	1 (reference)	0.17	1 (reference)	
F0-F2	105	23 (22)	5.8 (0.7–49.1)			
Indeterminate	42	14 (33)	3.4 (0.4–27.6)			

*BMI* body mass index, *ALT* alanine aminotransferase, *AST* aspartate aminotransferase, *ULN* upper limit of normal, *OR* odds ratio, *95% CI* 95 percent confidence interval, *n/a* not applicable

^a^ Smoking defined as more than one cigarette per week

^b^ Alcohol consumption defined as more than one glass per week

## Discussion

Spontaneous HBsAg loss in chronic HBV-infected patients is a phenomenon that is still not well understood. This study clearly shows that chronically HBV infected patients who lost HBsAg had lower sHA levels compared to patients who remained positive.

Though not reported in the literature previously, there are several arguments to explain this finding. First, sHA is a ligand of CD44 that is expressed in numerous cells, including liver sinus endothelial cells, neutrophils and regulatory T-cells (Tregs) [20, 21]. Data from experimental studies in other diseases have shown that there is an interplay between sHA and the immune system during chronic inflammation. On the one hand, studies in lung fibroblasts have shown that cytokines such as TNF-a activate the production of sHA [22]*.* Subsequent sHA-CD44 binding then promotes T-cell adhesion and migration to the endothelium to engage in an inflammatory process. On the other hand, there is also evidence that sHA stimulates the anti-inflammatory pathway of the immune system [23]*.* In an *in vitro* study, sHA binding to CD44 was correlated with a high suppressive activity of Tregs [23]*.*

Second, in chronic HBV-infected patients experiencing spontaneous HBsAg loss, early studies have shown that the inflammatory process in the liver declined after HBsAg clearance [12, 13]*.* Although the interplay between sHA, inflammation and HBsAg loss has to be further elucidated, we hypothesize that HBsAg loss dampens the immune system which in turn suppresses the synthesis of sHA leading to low sHA levels.

Several studies have investigated the value of sHA (or sHA as a component of the enhanced liver fibrosis [ELF] test) in patients with chronic HBV [8, 24–31]. However, these studies differ in several aspects from our study. First, until now studies in HBV patients have only focused on the role of sHA in the identification of liver fibrosis. Second, the study population of previous studies differed from our study, since previous studies either included only HBsAg positive patients or did not separate patients based on their HBsAg status (when HBsAg status of the included patients was not mentioned). Third, the cut-off value of the sHA concentration in our study is much lower than the cut-off values of other studies which varied between 52 and 300 ng/mL [25, 27]. Since we have shown that lower sHA values than previously reported are necessary to differentiate between patients with and without spontaneous HBsAg loss, this association could have gone unnoticed by others.

Since this was a cross-sectional study, we could not explore whether the sHA levels could be of predictive value to identify future candidates for spontaneous HBsAg loss. A prospective longitudinal study with multiple time points in which sHA levels would be determined and in which the moment of HBsAg loss is documented is needed to clarify this question. This would also allow for exploration of additional variables, which could be fitted into an algorithm, to identify chronic HBV patients who will experience spontaneous HBsAg loss in an early stage to avoid unnecessary antiviral therapy. The advantage of sHA is that it is easily accessible and available, relatively inexpensive and a small amount of serum is required for the assay.

In conclusion, serum HA levels are significantly lower in chronic HBeAg negative HBV patients who experience spontaneous HBsAg loss in comparison to those still being HBsAg positive. Further validation studies are needed to determine the predictive value of sHA with potential other variables in the natural history of chronic HBV.
